# Rethinking phenylalanine levels in phenylketonuria for optimal neurocognitive development beyond childhood

**DOI:** 10.3389/fped.2025.1488809

**Published:** 2025-06-19

**Authors:** Beatriz Câmara, Cristina Florindo, Cláudia Bandeira de Lima, Nélia Correia, Inês Fernandes, Manuela Batista, Ana Gaspar, Patrícia Janeiro

**Affiliations:** ^1^ Pediatric Department, Funchal Central Hospital, SESARAM-EPERAM, Funchal, Portugal; ^2^ Laboratory of Metabolism and Genetics, Department of Pharmaceutical Sciences and Medicines, Faculty of Pharmacy, University of Lisbon, Lisbon, Portugal; ^3^ Child Development Center, Pediatric Department, Santa Maria’s Hospital-Lisbon North University Hospital Center, EPE, Pediatric University Clinic, Faculty of Medicine, University of Lisbon, Lisbon, Portugal; ^4^ Lisbon Reference Center for Metabolic Diseases, Department of Pediatrics, Hospital Santa Maria, Unidade Local de Saúde Santa Maria, Lisbon, Portugal; ^5^ Faculdade de Medicina, Universidade de Lisboa, Lisbon, Portugal

**Keywords:** adolescent development, cognition, executive function, intelligence tests, phenylketonuria

## Abstract

**Introduction:**

Phenylketonuria (PKU) is an inborn error of phenylalanine (Phe) metabolism that disrupts neurotransmitter balance. Although early intervention has improved outcomes, neurocognitive challenges persist, particularly during adolescence. Metabolic control guidelines for patients aged >12 years differ between the European Union and the United States, with recommended blood Phe levels below 600 µM and 360 µM, respectively.

**Methods:**

This study evaluated the relationship between blood Phe levels, intelligence quotient (IQ), and executive functions using the Wechsler Intelligence Scale for Children-Third Edition and the d2 Test of Attention. Blood Phe levels were monitored longitudinally and summarized using the Index of Dietary Control (IDC), calculated as the mean of individual annual median Phe concentrations, both before and after 12 years of age.

**Results:**

The study included 14 early-treated PKU patients aged 12–17 years, all diagnosed through newborn screening programs. Participants maintained good metabolic control (IDC <360 µM) prior to 12 years of age, with a mean IDC of 302 µM. Higher IQ scores before the age of 12 years were observed only among patients with consistent dietary compliance. After that age, attentional performance declined in those who were noncompliant with dietary recommendations. Additionally, occasional elevations in blood Phe levels at the time of cognitive assessments were associated with poorer cognitive performance.

**Discussion:**

These findings underscore the detrimental effects of elevated Phe levels on executive functions during adolescence and highlight the need for larger studies to determine whether blood Phe levels between 360 and 600 µM are safe for patients aged >12 years.

## 1 Introduction

Phenylketonuria (PKU) is an autosomal recessive inborn error of metabolism caused by a deficiency of phenylalanine hydroxylase (PAH), leading to an increase of phenylalanine (Phe) levels in blood and tissues (1). PAH is encoded by the *PAH* gene, and to date, more than 900 different gene variants have been identified (2). Phe is an essential aromatic amino acid that is hydrolyzed to tyrosine (Tyr) in the liver by PAH, requiring tetrahydrobiopterin (BH4) as a cofactor (3).

Multiple pathophysiological mechanisms contribute to brain dysfunction in PKU. L-type amino acid transporter 1 is the predominant transporter for large-neutral amino acids at the blood-brain barrier and has a high affinity for Phe, leading to its increased concentration in the brain (4). Consequently, elevated Phe levels in patients with PKU result in excessive brain accumulation, which competitively inhibits the transport of other amino acids, such as Tyr and tryptophan (Trp), the precursors of dopamine and serotonin, respectively. As a result, the synthesis of both neurotransmitters and proteins is impaired (5–7). Glutamate transmission, critical for synaptic plasticity, learning, and memory, is also disrupted (8). Additionally, elevated Phe levels inhibit HMG-CoA reductase, impairing cholesterol synthesis, a key component of myelin membranes (9). Disruption of protein synthesis, along with the direct neurotoxic effects of Phe on myelin, leads to malformed dendritic trees, reduced neocortical synaptic density, and abnormal myelination (10). Neuroimaging studies have corroborated these histological findings by identifying white matter abnormalities in patients with PKU, which are directly associated with Phe levels and are partially reversible with adequate dietary control (11).

Treatment requires a lifelong phenylalanine-restricted diet, achieved through natural protein restriction and supplementation with Phe-free L-amino acid formulas containing appropriate blends of essential nutrients and amino acids (12). Treatment for PKU should be initiated as early as possible to mitigate Phe toxicity and promote optimal neuropsychological development (13). Since 2008, sapropterin, a synthetic analogue of the BH4 cofactor, has been available and shown to reduce blood Phe levels and enhance PAH stability in responsive patients (14). However, sapropterin responsiveness depends on genotype and the residual PAH activity. Other pharmacological treatments, such as pegvaliase, an enzyme substitution therapy, have recently been approved for late adolescents aged ≥16 years.

Initially, treatment protocols allowed discontinuation of the diet at approximately 6 years of age, under the assumption that significant neurodevelopment was complete (15). However, subsequent research demonstrated the impact of elevated blood Phe levels on neurocognition, leading to recommendations for extending dietary treatment to at least 12 years of age and, more recently, into adulthood (16).

Newborn screening for PKU was first implemented in Europe and the United States (US) in the 1960s through the evaluation of blood Phe levels using dried blood spot samples (17). This program has enabled early detection and treatment, significantly reducing the risk of severe neurological outcomes associated with untreated PKU, such as microcephaly, intellectual disability, and behavioral disorders (3).

Despite current practices aimed at reducing blood Phe levels, neurocognitive outcomes in patients with PKU remain suboptimal, particularly in those with poor metabolic control. Inadequate adherence to dietary treatment is commonly observed during adolescence due to factors, such as unpalatability and social limitations (14, 18).

To maintain adequate metabolic control, lifelong routine monitoring of blood Phe levels is essential (5). During childhood, the target blood Phe level is generally set below 360 µM; however, a global consensus on optimal Phe levels for adolescents and adults with PKU has not been established (19). Current European guidelines recommend maintaining blood Phe levels below 600 µM for patients aged >12 years (3), whereas US guidelines emphasize maintaining levels below 360 µM throughout life (20, 21).

Cognitive performance studies consistently show that children with PKU exhibit deficits, particularly in executive function, as well as a broad range of psychosocial and psychiatric difficulties compared with controls (19, 20, 22, 23). Notably, Fonnebeck et al. showed that the yearly mean Phe level is the most reliable biochemical predictor of adult intelligence quotient (IQ) (24). These findings suggest the importance of investigating neuropsychological outcomes across different ages and Phe thresholds to determine safe upper limits.

The primary aim of this study was to examine the association between dietary compliance, assessed through the Index of Dietary Control (IDC), and the neuropsychological profile—specifically cognition, information processing, and attention abilities—of a cohort of early-treated PKU adolescents continuously monitored since the neonatal period at a Metabolic Disease Reference Center in Lisbon. We used the Wechsler Intelligence Scale for Children–Third Edition (WISC-III) and the d2 Test of Attention (d2 test) to gather insights that could help clarify appropriate blood Phe thresholds (25–27).

## 2 Materials and methods

### 2.1 Patients

Patients with PKU (*N* = 18), aged 12–17 (mean age, 14) years, were recruited from the outpatient pediatric department of a Reference Center for Metabolic Diseases at a tertiary hospital in Lisbon. All participants were diagnosed through the National Newborn Screening (NBS) program and initiated a Phe-restricted diet within the first weeks of life (mean age of initiation: 14 days). Inclusion criteria comprised a complete clinical file, a comprehensive cognitive assessment, informed written consent from parents or legal guardians, and informed assent from patients. Data collected from clinical files included sex, age at diagnosis, age at diet initiation, PKU genotype, age at neuropsychological assessment, blood Phe levels throughout life, and ongoing treatment with sapropterin. Four patients were excluded due to incomplete clinical files. The study protocol was approved by the Institutional Ethics Committee (Ref. 241/19), and all procedures were performed in compliance with institutional guidelines. This research did not receive any specific funding from public, commercial, or non-profit sectors.

### 2.2 Neuropsychological assessment

Neuropsychological assessments were conducted by the hospital's team of neurodevelopmental psychologists, experienced in the intellectual evaluation of children and adolescents. Assessments were conducted between July 2019 and February 2020 using the WISC-III and the d2 test (25–27).

The WISC-III is a standardized measure of intellectual functioning for children aged 6–16 years. It assesses general intellectual ability through normative comparisons and provides insights into various cognitive domains impacting performance. The test comprises 13 subtests, combined to calculate several indices: Full-Scale IQ (FSIQ), Verbal IQ (VIQ), Performance IQ (PIQ), Verbal Comprehension Index (VCI), Perceptual Organization Index (POI), and Processing Speed Index (PSI). According to performance, children are classified as having average intelligence (FSIQ: 85–115), low average intelligence (FSIQ: 70–85), or below average intelligence (FSIQ: <70).

The d2 test is a psychological performance assessment designed to evaluate attention. It consists of 14 lines, each containing 47 characters comprising the letters “d” or “p” marked with one to four dashes above or below them. Participants are instructed to scan the lines and cross out all instances of the letter “d” with two dashes, disregarding other symbols. The test yields three primary outcomes reflecting processing speed, rule compliance, and quality of performance—critical components of concentration. Additionally, it assesses executive functions, such as mental processing speed, perceptual speed, working memory, and overall performance capacity. Metrics recorded include the total number of characters processed (TNC), concentration performance (CP), coefficient of variation of performance speed (CV), total correct guesses and relevant elements (TA), total correctly processed (TN-E), and error percentage (E%). Raw scores are converted into percentiles, facilitating comparisons among participants relative to a normative sample. Scores between the 25th and 75th percentiles are considered average.

All enrolled patients underwent comprehensive neuropsychological assessments during the study period. To avoid fatigue and stress, evaluations were split into two appointments spaced two to three weeks apart to ensure patient compliance. Final scores were derived by integrating data from both sessions, providing a comprehensive representation of each participant's cognitive functioning.

### 2.3 Data analysis

Blood Phe control values, registered from the first hospital appointment until the neuropsychological assessments, were collected for each patient with PKU. A total of 1,769 blood samples were analyzed, including 1,439 evaluations (range: 17–162 per patient) before 12 years of age and 330 evaluations (range: 1–86 per patient) after 12 years of age.

Patients were grouped based on their IDC, defined as the mean of the median blood Phe levels per year of life (28, 29). IDC was calculated for three time periods: IDC-A (throughout life), IDC-B (<12 years), and IDC-C (>12 years). Good dietary compliance was considered when IDC values were <360 µM.

### 2.4 Statistical analysis

Statistical analysis was conducted to examine the association between IDC and the different cognitive scores evaluated. Analyses were performed using GraphPad Prism version 8.0 (GraphPad Software, San Diego, CA). An unpaired *t*-test was used to compare WISC-III and d2 test results between patients with PKU. Each comparison was analyzed individually without assuming a consistent standard deviation, and no corrections for multiple comparisons were applied. A *p*-value <0.05 was considered statistically significant.

## 3 Results

### 3.1 Cohort of patients with PKU

We examined a cohort of 14 patients aged 12–17 years at the time of neuropsychological evaluation, of whom 13 were females. These patients had been followed since the neonatal period at a Reference Center for Metabolic Diseases and were diagnosed through the NBS program, presenting initial blood Phe levels ranging from 399.5 to 1501.2 µM, with a median value of 966.5 µM. The mean age at diagnosis was 8 days, and dietary treatment was initiated at a mean age of 14 days. Twelve different pathogenic variants were identified in the genotypes, with residual enzymatic activity ranging from 5% to 65% (BioPKU database; biopku.org). *PAH* gene variants are highly heterogeneous; 11 patients (85.7%) were compound heterozygotes. At the time of cognitive evaluation, two patients were receiving treatment with sapropterin. A detailed characterization of the cohort is summarized in Table 1.

**Table 1:** Characterization of patient cohort (*N* = 14).

Patient ID	Age (y)	Sex	Genotype (*PAH* gene)	Residual PAH activity	Diet (d)	Phe (µM)		
						IDC-A (mean ± SD)	IDC-B (mean ± SD)	IDC-C (mean ± SD)
I	14	F	*n.a./n.a.*	*n.a./n.a.*	27	163.1 ± 61.0	177.7 ± 47.6	46.1 ± 0.0
II	17	F	p.I65 T/p.R261Q	33%/44%	10	**575.4** ± **251.4**	**447.0** ± **131.3**	**832.1** ± **237.2**
III	16	F	p.A300S/p.D222X	65%/*n.a.*	18	251.2 ± 43.1	251.0 ± 47.9	252.1 ± 23.7
IV	14	F	p.R243Q/p.R261Q	14%/44%	8	**399.3** ± **159.3**	334.6 ± 95.2	**658.0** ± **84.1**
V	17	F	p.R241H/p.R261Q	23%/44%	21	250.9 ± 75.0	238.4 ± 71.0	275.9 ± 76.6
VI	15	F	p.L249F/p.P281l	51%/2%	15	322.5 ± 67.8	294.5 ± 43.5	**406.5** ± **57.3**
VII	15	F	p.R261Q/p.V388M	44%/28%	22	174.4 ± 50.5	159.0 ± 47.9	220.5 ± 21.7
VIII	12	F	p.V388M/IVS10-11G > A	28%/5%	12	**427.5** ± **130.3**	**419.4** ± **132.5**	**524.2** ± **0.0**
IX	16	F	p.R243Q/p.V388M	14%/28%	8	**472.4** ± **273.5**	304.8 ± 103.8	**807.7** ± **185.0**
X	14	F	p.R261Q/p.V388M	44%/28%	9	264.6 ± 84.0	241.1 ± 75.8	358.3 ± 35.7
XI	14	F	p.V388M/p.D129G	28%/*n.a.*	15	257.6 ± 51.3	249.8 ± 52.6	288.8 ± 29.6
XII	16	F	p.L249F/p.A309V	51%/42%	15	287.5 ± 47.2	268.3 ± 35.6	333.7 ± 38.8
XIII	14	F	IVS10-11G > A/IVS10-11G > A	5%/5%	9	**420.8** ± **184.6**	**380.7** ± **173.1**	**581.4** ± **135.4**
XIV	12	M	IVS10-11G > A/IVS10-11G > A	5%/5%	8	**485.9** ± **167.8**	**462.5** ± **158.0**	**719.7** ± **0.0**
*Mean*	*14*	*–*	*–*	*–*	*14*	*339.5*	*302.1*	*450.4*
**±** *SD*	**±** *2*	*–*	*–*	*–*	**±** *6*	**±** *120.1*	**±** *91.8*	**±** *231.1*

IDC values >360 µM are highlighted (bold).

Age (years), Age at the time of psychological assessment, in years; residual PAH enzyme activity according to BioPKU database; Diet (d), start of dietary treatment in days; IDC, index of dietary control calculated as the mean of the median blood Phe level by year; IDC-A, throughout life until neuropsychological assessment; IDC-B, <12 years of age; IDC-C, 12 years of age until the neuropsychological assessment.

SD, Standard deviation; *n.a.*, not available; F, female; M, male.

### 3.2 Neuropsychological assessment

The FSIQ, measured by the WISC-III test, ranged from 51 to 111 points, with an average score of 87 points. Most patients (9/14) demonstrated average intelligence, while three patients exhibited low average intelligence, and two were classified as having below average intellectual development.

Subscale analyses revealed performance skills scores of 49–118 (mean: 88) points and verbal scores of 58–126 (mean: 90) points.

Regarding the d2 test, total efficacy scores varied from 1 to 80 (mean: 50) points, while concentration index scores ranged from 1 to 90 (mean: 54) points. Detailed results from the neuropsychological assessment (WISC-III and d2 Test) are provided in Supplementary Table S1.

### 3.3 Comparative analysis of neurocognitive outcomes

**Figure 1:**
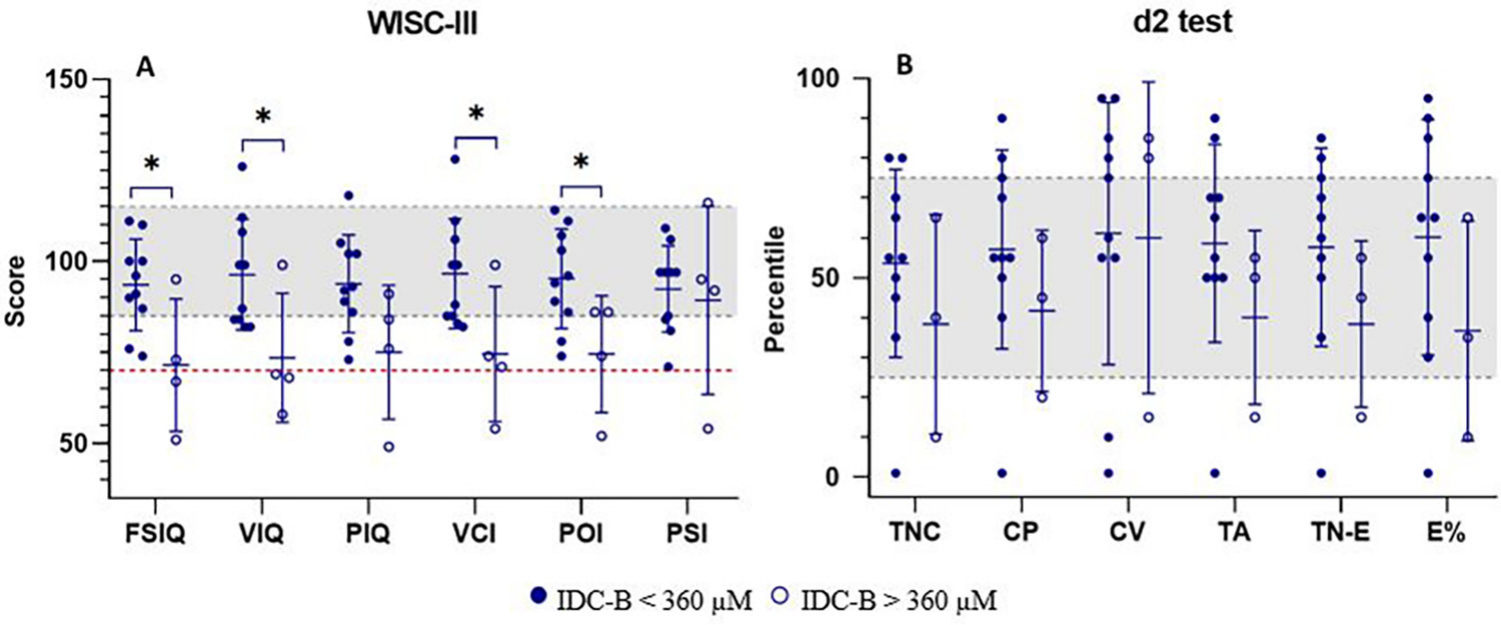
Neuropsychological assessment of patients with PKU grouped according to their index of dietary control (IDC) before 12 years of age. **(A)** Wechsler intelligence scale for children – third edition (WISC-III) scores; **(B)** percentile scores on the d2 test of attention (d2 test). Patients with IDC-A <360 µM (filled circles, *N* = 10) and IDC-A >360 µM (open circles, *N* = 4 for WISC-III; *N* = 3 for d2 test). Statistically significant differences between groups (*p* < 0.05): FSIQ (*p* = 0.022), VIQ (*p* = 0.032), VCI (*p* = 0.038), POI (*p* = 0.031). The gray shaded area indicates the normative performance range for IQ scores **(A)** and d2 test percentiles **(B)**. The red dashed line in **(A)** denotes an IQ score of 75, commonly used as the threshold for intellectual impairment. CP, concentration performance; CV, coefficient of variation of performance speed; E%, error percentage; FSIQ, full-scale IQ; PIQ, performance IQ; POI, perceptual organization index; PSI, processing speed index; TA, total number of correct guesses and relevant elements; TC-E, total correct for errors; TNC, total number of characters processed; VCI, verbal comprehension; VIQ, verbal IQ.

To evaluate the influence of blood Phe levels during the first 12 years of life on neurocognitive outcomes, patients were subdivided into two groups based on their IDC: below or above 360 µM. Neurocognitive outcomes assessed by WISC-III and d2 Test scores are presented in Figure 1.

Statistically significant differences were observed in several IQ test components (FSIQ, *p* = 0.022; VIQ, *p* = 0.032; VCI, *p* = 0.038; POI, *p* = 0.031). These findings are consistent with the well-established association between elevated blood Phe levels (>360 µM) and lower IQ scores during the critical neurodevelopmental period up to 12 years of age.

**Figure 2:**
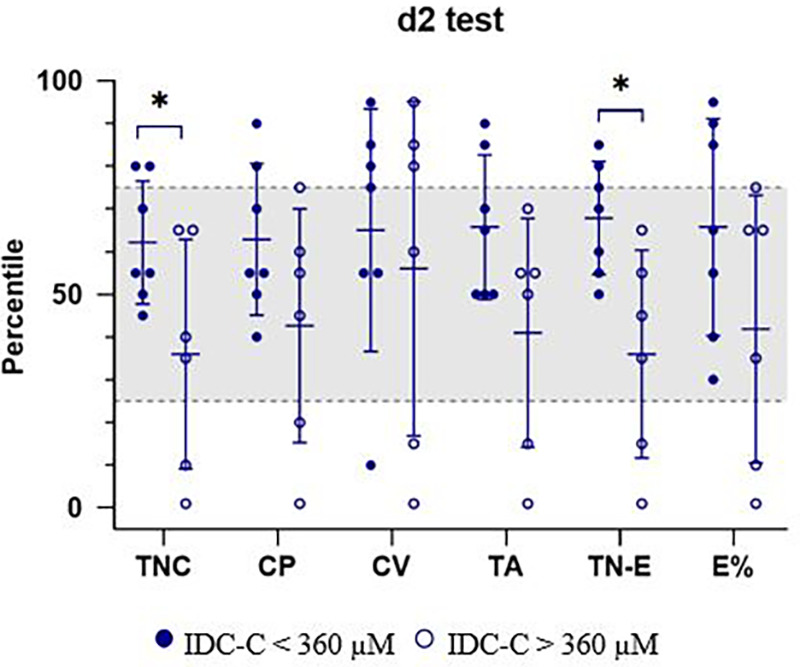
d2 test of attention (d2 test) assessment of patients with PKU grouped according to their index of dietary control after 12 years of age, up to the time of neuropsychological evaluation. Patients with IDC-C <360 µM (filled circles, *N* = 7) and IDC-C >360 µM (open circles, *N* = 6). Statistically significant differences between groups (*p* < 0.05): TNC (*p* = 0.047), TN-E (*p* = 0.012). The gray shaded area indicates the normative percentile range for d2 test performance. CP, concentration performance; CV, coefficient of variation of performance speed; E%, error percentage; TA, total number of correct guesses and relevant elements; TC-E, total correct for errors; TNC, total number of characters processed.

It is noteworthy that statistically significant differences in d2 Test outcomes (TNC, *p* = 0.047; TN-E, *p* = 0.012) were detected only among patients with IDC >360 µM after 12 years of age. Further details are depicted in Figure 2.

**Figure 3:**
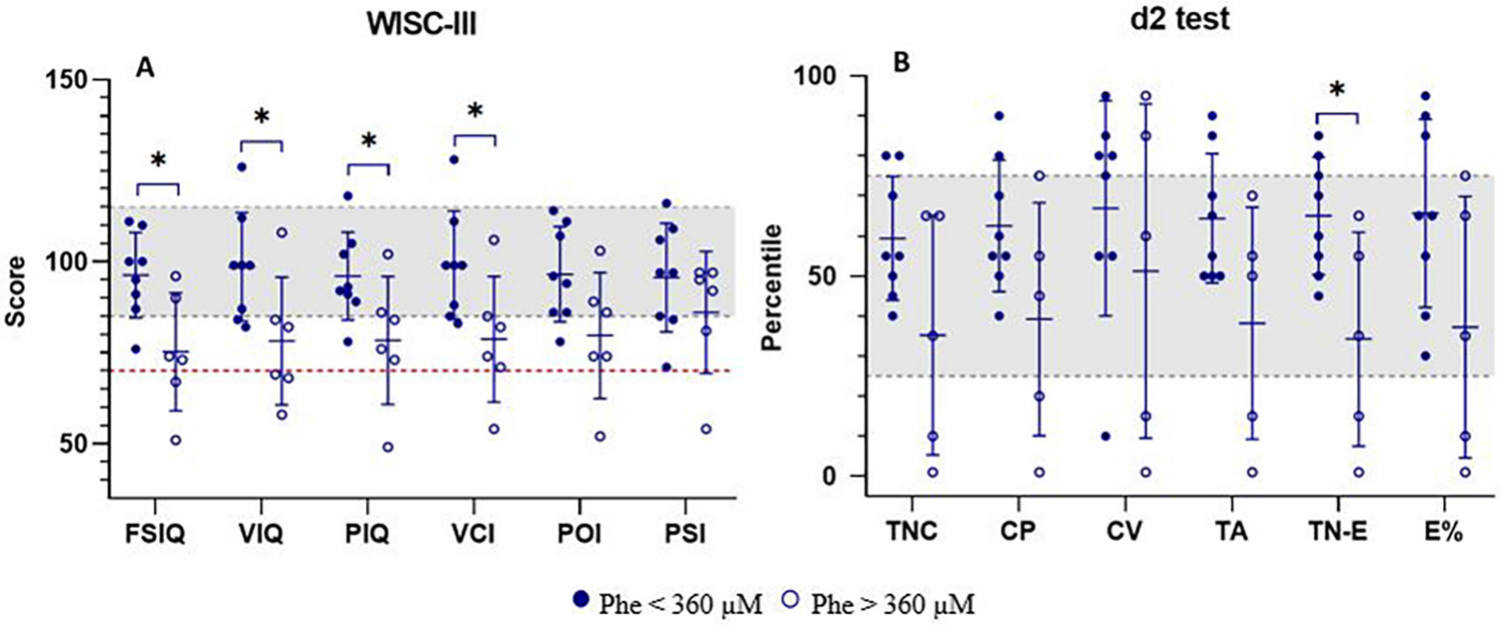
Neuropsychological assessment of patients with PKU grouped according to the absolute value of blood Phe level at the time of cognitive assessment. **(A)** Wechsler intelligence scale for children – third edition (WISC-III) scores; **(B)** percentile scores on the d2 Test of attention (d2 test). patients with Phe <360 µM (filled circles, *N* = 8) and Phe >360 µM (open circles, *N* = 6 for WISC-III; *N* = 5 for d2 test). Statistically significant differences between groups (*p* < 0.05): FSIQ (*p* = 0.015), VIQ (*p* = 0.037), PIQ (*p* = 0.045), VCI (*p* = 0.036), TN-E (*p* = 0.020). The gray shaded area indicates the normative performance range for IQ scores **(A)** and d2 test percentiles **(B)**. The red dashed line in **(A)** denotes an IQ score of 75, commonly used as the threshold for intellectual impairment. CP, concentration performance; CV, coefficient of variation of performance speed; E%, error percentage; FSIQ, full-scale IQ; PIQ, performance IQ; POI, perceptual organization index; PSI, processing speed index; TA, total number of correct guesses and relevant elements; TC-E, total correct for errors; TNC, total number of characters processed; VCI, verbal comprehension index; VIQ, verbal IQ.

Given patients' complaints regarding concentration difficulties and emotional instability (personal communication) during periods of elevated blood Phe, we also analyzed the potential impact of absolute blood Phe values (range: 410.0–933.9 µM) at the time of cognitive assessment. Results are shown in Figure 3.

These findings corroborate patients' subjective experiences, with significant differences observed between groups in the TN-E d2 score (*p* = 0.020) and multiple IQ measures (FSIQ, *p* = 0.015; VIQ, *p* = 0.037; PIQ, *p* = 0.045; VCI, *p* = 0.036).

## 4 Discussion

There is consensus among PKU scientific boards that patients aged >12 years with blood Phe levels >600 μM should be treated, and those with blood Phe levels below 360 μM are considered within the recommended target range. However, debate remains regarding the appropriate management of patients whose blood Phe levels fall between 360 and 600 μM.

In this context, the European guideline recommends maintaining blood Phe levels below 360 μM as the upper target for the first 12 years of life to prevent cognitive impairment, and thereafter permits a broader range up to 600 μM (30). In contrast, the American College of Medical Genetics and Genomics advocates for a lifelong target limit of 360 μM (16). Therefore, it is of paramount importance to investigate the neuropsychological outcomes of patients aged >12 years with blood Phe levels below and above the accepted target level of 360 μM, to help clarify the upper safe limits.

Impairments in neurocognitive performance, particularly lower IQ scores, are associated with Phe levels exceeding 360 μM before the age of 12 years. Canton et al. (5) conducted a systematic review of studies evaluating neuropsychological performance in children and adolescents with early-treated PKU. Half of the included studies employed WISC tests to assess intellectual functioning and demonstrated that, although the mean FSIQ remained within the average range, it was significantly lower compared to controls.

Notably, in our study, among the four patients with PKU with an individual IDC-A >360 μM (380.7 ± 173.1 to 462.5 ± 158.0), below-average intellectual development (FSIQ <70) was observed only in the two patients bearing the most severe genotype, the IVS10-11G >A mutation in homozygosity, thus highlighting the influence of genotype on metabolic phenotype.

It is well known that crucial cognitive functions, such as inhibition, working memory, and cognitive flexibility, emerge during early childhood but continue to develop and strengthen throughout adolescence. A recently published review by Thomas et al. (31), which included a meta-analysis of 46 studies investigating the impact of metabolic control on cognition, neurophysiology, and well-being in patients with PKU, emphasized the cognitive benefits of lower Phe levels, particularly in the domains of attention, executive functions, and processing speed. Similarly, Diamond et al. (32) assessed the executive functions of children with PKU over 4 years and found impairments in working memory and inhibition when Phe levels were three to five times above the normal range.

Our findings are consistent with these reports. We observed that the Phe levels exceeding 360 μM, even after good metabolic control during the first 12 years of life, negatively impact higher intellectual functions, particularly accuracy. However, it should be noted that the test administered primarily evaluates individual attention capacity and executive functions, parameters that may be influenced by age.

Moreover, our results showed that occasional periods of elevated blood Phe levels also impact cognitive processes and intellectual capacity (33). These findings align with those reported by Anastasoaie et al. (3), who demonstrated that, although mean lifetime blood Phe levels were significantly correlated with FSIQ, long-term variability in Phe levels among well-controlled children had an even greater impact on cognitive outcomes. They concluded that when the mean Phe levels exceeded the recommended range, variability further exacerbated the deleterious effects of Phe exposure.

European guidelines suggest that neuropsychological assessments should be conducted only when clinically justified during childhood, with routine evaluations recommended at 12 and 18 years of age to address changes in treatment targets and support the social transition to adulthood (30). Nevertheless, based on our findings, it may be pertinent to advocate for a standardized and comprehensive neurodevelopmental evaluation earlier in the child's developmental trajectory, to identify patients at risk for adverse outcomes and to enable timely management and therapeutic interventions.

### 4.1 Study limitations

Education, socioeconomic status, general health, and well-being are variables that may affect cognitive abilities and should be considered in patients' neuropsychological assessments. The cross-sectional design and the limited sample size are the two main limitations of our study, which hinders the ability to establish absolute cause-effect relationships.

## 5 Conclusions

In summary, our findings align with previous reports, supporting the notion that elevated Phe levels adversely impact cognitive functions throughout life, with detrimental effects on IQ during early developmental stages and on central executive functions during adolescence, even after an initial period (<12 years) of good metabolic control. Current evidence remains insufficient to assert that the Phe levels in the range of 360–600 μM are safe for patients aged >12 years. Given the limitations of the present study, further research is warranted to recommend a stricter upper target for Phe levels.

It is therefore of paramount importance to implement a comprehensive, standardized neuropsychological test battery across international PKU centers. Larger sample sizes and extended follow-up periods are essential to better identify disease-independent individual factors—whether environmental or neurobiological—that may modulate the impact of biochemical alterations. Multicenter studies will not only enhance our understanding of PKU-related neurodevelopmental pathophysiology but also contribute to the standardization of neurocognitive surveillance and the identification of more effective therapeutic interventions.

## Data Availability

The original contributions presented in the study are included in the article/Supplementary Material, further inquiries can be directed to the corresponding author.
