# Supplementary material for: Rethinking phenylalanine levels in phenylketonuria for optimal neurocognitive development beyond childhood
Source: Front Pediatr. 2025 Jun 19;13:1488809. doi: 10.3389/fped.2025.1488809 (PMC12222274; doi:10.3389/fped.2025.1488809)
Supplement: Supplementary file 1 [file Table1.pdf]

Table S1. Neuropsychological assessment of PKU patients enrolled in the study.

Patient ID	WISC-III						d2 test of attention				
	FSIQ	VIQ	PIQ	VCI	POI	PSI	TNC	CP	CV	TN-E	E%
I	87	87	93	88	96	97	45	55	85	80	65
II	95	99	91	99	86	116	40	60	80	45	65
III	110	112	105	111	107	97	80	40	75	75	30
IV	74	84	73	82	74	81	1	1	1	1	1
V	76	82	78	83	78	85	50	50	55	50	55
VI	96	108	86	106	89	97	65	75	95	65	65
VII	100	99	102	99	111	71	55	55	95	55	40
VIII	73	69	84	74	86	95	65	45	15	55	10
IX	90	82	102	85	103	97	35	55	60	35	75
X	111	126	92	128	94	106	70	80	55	70	85
XI	100	84	118	85	114	84	55	70	10	60	90
XII	91	99	89	99	86	109	80	90	80	85	95
XIII	67	68	76	71	74	92	10	20	85	15	35
XIV	51	58	49	54	52	54	*n.a.*	*n.a.*	*n.a.*	*n.a.*	*n.a.*
Mean ± SD	*87 ± 16*	*90 ± 18*	*88 ± 16*	*90 ± 18*	*89 ± 16*	*92 ± 15*	*50 ± 23*	*54 ± 23*	*61 ± 31*	*53 ± 24*	*55 ± 29*

Note: Average intelligence: score 85 – 115; low average intelligence: score 70 – 85; below average intellectual development: score < 70. Normal d2 test performance: percentile 25th – 75th.

Abbreviations: FSIQ - Full-Scale IQ (intelligence quotient); VIQ - Verbal IQ; PIQ - Performance IQ; VCI - Verbal Comprehension Index; POI - Perceptual Organization Index; PSI - Processing Speed Index; TNC - Total Number of Characters processed; CP - Concentration Performance; CV - Coefficient of Variation of performance speed; TA - Total number of correct guesses and relevant elements; TN-E - Total correctly processed; E% - Error Percentage. SD - standard deviation; *n.a.* - not available.
